# Validation of the Cardiac Arrest Survival Postresuscitation In-hospital (CASPRI) score in an East Asian population

**DOI:** 10.1371/journal.pone.0202938

**Published:** 2018-08-23

**Authors:** Chih-Hung Wang, Wei-Tien Chang, Chien-Hua Huang, Min-Shan Tsai, Ping-Hsun Yu, Yen-Wen Wu, Wen-Jone Chen

**Affiliations:** 1 Department of Emergency Medicine, National Taiwan University Hospital, Taipei, Taiwan; 2 Department of Emergency Medicine, College of Medicine, National Taiwan University, Taipei, Taiwan; 3 Department of Emergency Medicine, Taipei Hospital, Ministry of Health and Welfare, New Taipei City, Taiwan; 4 Departments of Internal Medicine and Nuclear Medicine, National Taiwan University Hospital and National Taiwan University College of Medicine, Taipei, Taiwan; 5 Department of Nuclear Medicine and Cardiology Division of Cardiovascular Medical Center, Far Eastern Memorial Hospital, New Taipei City, Taiwan; 6 National Yang-Ming University School of Medicine, Taipei, Taiwan; 7 Division of Cardiology, Department of Internal Medicine, National Taiwan University Hospital and National Taiwan University College of Medicine, Taipei, Taiwan; University of Bern, University Hospital Bern, SWITZERLAND

## Abstract

**Background:**

The Cardiac Arrest Survival Postresuscitation In-hospital (CASPRI) score is a useful tool for predicting neurological outcome following in-hospital cardiac arrest (IHCA), and was derived from a cohort selected from the Get With The Guidelines-Resuscitation registry between 2000 and 2009 in the United States. In an East Asian population, we aimed to identify the factors associated with outcomes of resuscitated IHCA patients and assess the validity of the CASPRI score.

**Methods:**

A retrospective study was conducted in a single centre in Taiwan. Patients with IHCA between 2006 and 2014 were screened.

**Results:**

Among the 796 included patients, 94 (11.8%) patients achieved neurologically intact survival. Multivariable logistic regression analyses identified factors significantly associated with neurological outcome. Six of these factors were also components of the CASPRI score, including duration of resuscitation, neurological status before IHCA, malignant disease, initial arrest rhythms, renal insufficiency and age. In univariate logistic regression analysis, the CASPRI score was significantly associated with neurological outcome (odds ratio [OR]: 0.83, 95% confidence interval [CI]: 0.80–0.87); the area under the receiver operating characteristics curve was 0.79 (95% CI: 0.74–0.84).

**Conclusion:**

In this retrospective study conducted in a single centre at Taiwan, we identified the common prognosticators of IHCA shared by both East Asian and Western societies. As a composite prognosticator, CASPRI score predicts outcomes with excellent accuracy among successfully resuscitated IHCA patients in an East Asian population. This tool allows accurate IHCA prognostication in an East Asian population.

## Introduction

In the United States, approximately 209,000 patients experience in-hospital cardiac arrest (IHCA) each year [1], corresponding to an incidence of 3–6 events per 1000 hospital admissions [2]. Despite continuing efforts to improve the “chain of survival” protocol, patient outcomes after IHCA remain poor. Approximately 24% of IHCA patients survive to hospital discharge; among these patients, approximately 14% experience significant neurological disability [1].

Many of these IHCA survivors may require care in nursing homes and long-term care facilities following discharge, which places a significant burden on the family and society [3]. Do-not-resuscitate (DNR) orders may be used to withhold cardiopulmonary resuscitation (CPR) from patients who are unlikely to benefit from CPR or for whom CPR is inconsistent with their personal preferences. However, to discuss a plan for CPR preemptively, before patients actually experience IHCA, may be difficult in an East Asian population.

It is reported that in an East Asian population, even if patients have already consented to DNR orders, when they suffer from severe sepsis/septic shock, they may still receive more aggressive interventions, such as arterial and central venous catheterization, than those without DNR orders despite that the mortality is as high as 90% [4]. Because of the filial piety and family-centred end-of-life care stressed by the Confucianism [5], families may wish to treat the patient as they can even if the predicted outcome is bleak.

Because of the unique perspective about end-of-life care in East Asia [5], a prediction model for patients resuscitated from the initial IHCA may aid in such end-of-life decisions. Chan et al. [6] developed the Cardiac Arrest Survival Postresuscitation In-hospital (CASPRI) score to predict the outcomes of patients who experience IHCA and achieve return of spontaneous circulation (ROSC). The CASPRI score was derived from the large database of Get With The Guidelines-Resuscitation (GWTG-R) in the United States, which included 42957 patients from 551 hospitals. The score is calculated by summing points from 11 variables, including age, initial arrest rhythm or elapsed time to defibrillation, baseline neurological status, arrest location, CPR duration, presence of mechanical ventilation, renal insufficiency, hepatic insufficiency, sepsis, malignant disease, and hypotension prior to the arrest.

In the internal validation cohort of the original study, the CASPRI score had excellent discrimination and calibration. The CASPRI score demonstrates the ability to stratify patients, who achieved ROSC following the initial IHCA, into a wide range of probabilities of favourable neurological outcome. Patients in the worst category have only a 2.8% probability of neurologically intact survival, for whom the discussion of DNR orders may be appropriate. Because the proportion of Asian patients was low in the GWTG-R database [7, 8], in this study, we first evaluated the factors associated with neurological outcome of resuscitated IHCA patients in our cohort and then evaluated the validity of the CASPRI score in an East Asian population.

## Materials and methods

### Setting

This retrospective cohort study was performed in the tertiary medical centre National Taiwan University Hospital (NTUH). NTUH has 2,600 beds, including 220 beds in intensive care units (ICUs). This study was conducted in accordance with the Declaration of Helsinki. The Research Ethics Committee of the NTUH approved this study (reference number: 201706012RINB) and waived the requirement for informed consent before data collection. According to hospital policy, a resuscitation team is activated when a cardiac arrest event occurs in the general wards. A resuscitation team consists of a senior resident, four junior residents, a respiratory therapist, a head nurse, and two ICU nurses. In the ICUs, a resuscitation team is not mobilised for cardiac arrest events; instead, resuscitation is performed by the ICU staff where the event occurs and by staff from neighbouring ICUs.

At NTUH, after successful resuscitation for IHCA, neuroprognostication is performed using a daily assessment of brainstem reflex, as well as electroencephalography on the 3rd and 7th days. In Taiwan, before 2015, legislation did not allow withdrawal of life-sustaining therapy for post-cardiac arrest patients. Therefore, the results of neuroprognostication could not aid in discussions about whether life-sustaining therapy should be withdrawn, but could only aid surrogates and clinicians to determine whether further aggressive life-sustaining therapy should be added.

### Participants

The Centre of Quality Management of NTUH identified IHCA patients using the procedure code of CPR, which was used for insurance reimbursement. Patients who experienced IHCA at NTUH from 2006 to 2014 were screened. Patients who met the following criteria were included in the study: (1) aged 18 years or older, (2) documented absence of pulse with performance of chest compressions for at least 2 minutes, (3) no documentation of a DNR order before arrest, and (4) achievement of sustained ROSC. Sustained ROSC was defined as ROSC lasting ≥ 20 minutes without resumption of chest compressions. If multiple cardiac arrest events occurred in a single patient during hospitalization, only the first event was recorded. Patients who experienced cardiac arrest related to major trauma were excluded from the study.

### Data collection and outcome measures

The following information was recorded for each patient: age, gender, comorbidities, variables recorded on the Utstein template [9], and critical interventions implemented at the time of cardiac arrest. Duration of CPR was defined as the time interval from the first chest compression initiated by the resuscitation team or ICU members to the termination of resuscitation efforts, either due to sustained ROSC or declaration of death. The CASPRI score was calculated for each patient according to S1 Table. The definition of each independent variable in our study was the same as reported by Chan et al. [6] in the original development and internal validation study.

The primary outcome was favourable neurological status at hospital discharge. Favourable neurological status was defined as a score of 1 or 2 on the Cerebral Performance Category (CPC) scale [10], as reported by Chan et al. [6] The CPC score is a validated 5-point scale of neurological disability (1, good cerebral performance; 2, moderate cerebral disability; 3, severe cerebral disability; 4, coma/vegetative state; 5, death). Patients with a CPC score of 1 or 2 had sufficient cerebral function to live independently. The CPC score was retrospectively determined by two research assistants who reviewed medical records for each patient.

### Statistical analysis

Data were analysed using R 3.3.1 software (R Foundation for Statistical Computing, Vienna, Austria). Categorical data were expressed as counts and proportions; continuous data were expressed as median and interquartile ranges. Categorical variables were compared using Fisher’s exact test; continuous variables were examined using Wilcoxon’s rank-sum test. A two-tailed *p*-value of <0.05 was considered statistically significant.

First, we attempted to identify factors associated with the primary outcome in our cohort to identify the common prognosticators shared by our study the CASPRI score. Multivariable logistic regression analyses were performed to examine the associations between independent variables and the outcome. The odds ratio (OR) was selected as the outcome measure. All available independent variables without missing data were considered in the regression model, regardless of whether they were significantly associated with the outcome by univariate analysis. The stepwise variable selection procedure (with iterations between the forward and backward steps) was applied to select the final regression model. Significance levels for entry and to stay were set at 0.15 to avoid exclusion of potential candidate variables. The final regression model was selected by excluding individual variables with a *p*-value >0.05 until all regression coefficients were statistically significant. We assessed the goodness-of-fit of the fitted regression model using area under the receiver operating characteristics curve (AUROCC), adjusted generalised *R*^2^, and the Hosmer-Lemeshow goodness-of-fit test.

Secondly, the CASPRI score was analysed as a continuous variable against the primary outcome using univariate logistic regression analysis. In the original study, the CASPRI score was derived from the GWTG-R database during the time period from 2000 to 2009 [6]. The generalizability of the CASPRI score to IHCA patients after the implementation of 2010 resuscitation guidelines [11] was not known. Therefore, the AUROCC of the CASPRI score was calculated for the whole cohort and for two subgroups defined by the year IHCA occurred, either ≦2010 or >2010.

## Results

As shown in S1 Fig, between 2006 and 2014, 1,538 adult patients at NTUH received chest compressions for ≥2 min. Of these, 13 patients were excluded because of trauma-related cardiac arrest. Among the remaining 1,525 patients, 796 patients achieved sustained ROSC and were enrolled in our study for further analysis.

The median age of the patients was 66.8 years; 466 (58.5%) patients were male. There were 358 (45.0%) patients receiving CPR during or before year 2010. The majority (80.8%) of initial rhythms were non-shockable rhythms, including pulseless electrical activity and asystole. The median CPR duration was 15 minutes. A total of 388 events of cardiac arrest (48.7%) occurred in the ICUs, and 408 events (51.3%) occurred on the general wards, including both monitored and nonmonitored beds. Ninety-four (11.8%) patients survived to hospital discharge with favourable neurological status. The median CASPRI score was 24 points; the score was significantly lower in patients with favourable neurological status at hospital discharge.

All independent variables listed in Tables 1 and 2, except the CASPRI score, were included in the regression analysis for variable selection. Table 3 displays the ORs of 12 factors significantly associated with the primary outcome. Among these were six factors that are also components of the CASPRI score: CPR duration, neurological status before IHCA, malignant disease, initial arrest rhythms, renal insufficiency and age. The AUROCC was 0.86 (95% confidence interval [CI]: 0.83–0.90) for the final regression model presented in Table 3.

**Table 1:** Baseline characteristics of study patients by neurological status at hospital discharge.

Variables	All patients (n = 796)	Patients with favourable neurological outcome at hospital discharge (n = 94)	Patients without favourable neurological outcome at hospital discharge (n = 702)	*p*-value
Age (year), median (IQR^a^)	66.8 (54.7–77.1)	63.5 (50.0–73.8)	67.2 (55.5–77.9)	0.035
Male, n (%)	466 (58.5)	70 (74.5)	396 (56.4)	0.001
Neurological status 24 h before cardiac arrest, n (%)				0.001
CPC^b^ 1	35 (4.4)	6 (6.4)	29 (4.1)	
CPC 2	312 (39.2)	52 (55.3)	260 (37.0)	
CPC 3	324 (40.7)	31 (33.0)	293 (41.7)	
CPC 4	125 (15.7)	5 (5.3)	120 (17.1)	
Comorbidities, n (%)				
Respiratory insufficiency	579 (72.7)	58 (61.7)	521 (74.2)	0.013
Renal insufficiency	357 (44.8)	31 (33.0)	326 (46.4)	0.015
Arrhythmia	157 (19.7)	22 (23.4)	135 (19.2)	0.335
Diabetes mellitus	287 (36.7)	34 (36.2)	253 (36.0)	1
Hypotension	186 (23.4)	18 (19.1)	168 (23.9)	0.363
Heart failure, prior admission	127 (16.0)	22 (23.4)	105 (15.0)	0.050
Heart failure, this admission	164 (20.6)	30 (31.9)	134 (19.1)	0.006
Myocardial infarction, prior admission	34 (4.3)	9 (9.6)	25 (3.6)	0.013
Myocardial infarction, this admission	106 (13.3)	26 (27.7)	80 (11.4)	<0.001
Metabolic or electrolyte abnormality	139 (17.5)	12 (12.8)	127 (18.1)	0.247
Sepsis	78 (9.8)	4 (4.3)	74 (10.5)	0.062
Pneumonia	270 (33.9)	21 (22.3)	249 (35.5)	0.011
Metastatic or hematologic malignant disease	163 (20.5)	2 (2.1)	161 (22.9)	<0.001
Baseline depression in CNS^c^ function	231 (29.0)	18 (19.1)	213 (30.3)	0.029
Hepatic insufficiency	153 (19.2)	12 (12.8)	141 (20.1)	0.096
Acute stroke	41 (5.2)	2 (2.1)	39 (5.6)	0.214
Cardiopulmonary resuscitation during or before 2010, n (%)	358 (45.0)	31 (33.0)	327 (46.6)	0.015

^a^ IQR, interquartile range.

^b^ CPC, cerebral performance score

^c^ CNS, central nervous system

**Table 2:** Features and interventions of cardiac arrest events by neurological status at hospital discharge.

Variables	All patients (n = 796)	Patients with a favourable neurological outcome at hospital discharge (n = 94)	Patients without a favourable neurological outcome at hospital discharge (n = 702)	*p*-value
Initial cardiac arrest rhythm, n (%)				<0.001
Pulseless electrical activity	268 (33.7)	25 (26.6)	243 (34.6)	
Asystole	375 (47.1)	28 (29.8)	347 (49.4)	
VF^a^/ Pulseless VT^b^	153 (19.2)	41 (43.6)	112 (16.0)	
CPR^c^ duration (min), median (IQR^d^)	15 (7–26.3)	8 (5–15)	15 (8–29)	<0.001
Time to defibrillation (min),^e^ median (IQR)	2 (0–9)	1 (0–2)	2.5 (0–12)	0.009
Arrest location, n (%)				0.190
Intensive care unit	388 (48.7)	54 (57.4)	334 (47.6)	
Monitored unit	91 (11.4)	10 (10.6)	81 (11.5)	
Nonmonitored unit	317 (39.8)	30 (31.9)	287 (40.9)	
Arrest at night, n (%)	252 (31.7)	33 (35.1)	219 (31.2)	0.479
Arrest on weekend, n (%)	234 (29.4)	26 (27.7)	208 (29.6)	0.810
Interventions begun prior to arrest, n (%)				
Mechanical ventilation	156 (19.6)	15 (16.0)	141 (20.1)	0.407
Intravenous vasopressors	311 (39.1)	34 (36.2)	277 (39.5)	0.575
Intravenous antiarrhythmics	75 (9.4)	16 (17.0)	59 (8.4)	0.013
Pulmonary artery catheter	10 (1.3)	4 (4.3)	6 (0.9)	0.022
Hemodialysis	66 (8.3)	6 (6.4)	60 (8.5)	0.556
Intra-aortic balloon pumping	10 (1.3)	3 (3.2)	7 (1.0)	0.103
CASPRI^f^ score, median (IQR)	24 (19–28)	17 (12.3–22)	25 (20–28)	<0.001

^a^ VF, ventricular fibrillation

^b^ VT, ventricular tachycardia

^c^ CPR, cardiopulmonary resuscitation

^d^ IQR, interquartile range

^e^ For arrests due to VF or pulseless VT

^f^ CASPRI score, Cardiac Arrest Survival Post-resuscitation In-hospital score

**Table 3:** Multiple logistic regression model with favourable neurological outcome at hospital discharge as the dependent variable.

Independent variable^a^	Odds ratio	95% confidence interval	*p* value
Pulmonary artery catheter in place at time of arrest	9.85	2.00–48.65	0.005
Intravenous antiarrhythmics in place at time of arrest	2.93	1.38–6.24	0.005
**Favourable neurological status 24 h before cardiac arrest**^**b**^	2.88	1.72–4.85	<0.001
Myocardial infarction, this admission	2.73	1.43–5.22	0.002
**VF**^**c**^**/ Pulseless VT**^**d**^	2.58	1.50–4.47	0.001
Male	1.89	1.08–3.30	0.025
**Age (year)**	0.98	0.96–0.99	0.009
**CPR**^**e**^ **duration (min)**	0.94	0.91–0.96	<0.001
Cardiopulmonary resuscitation during or before 2010	0.56	0.32–0.97	0.037
**Renal insufficiency**	0.45	0.26–0.77	0.004
Baseline depression in CNS^f^ function	0.29	0.15–0.57	<0.001
**Metastatic or hematologic malignant disease**	0.08	0.02–0.32	<0.001

Goodness-of-fit assessment: n = 796, adjusted generalised *R*^*2*^ = 0.4; the estimated area under the receiver operating characteristics curve = 0.86, with 95% confidence interval 0.83–0.90; and the Hosmer and Lemeshow goodness-of-fit Chi-Squared test *p* = 0.71.

^a^ The display of independent variables is arranged by descending order of odds ratio. The bold-typed variables were also components of Cardiac Arrest Survival Post-resuscitation In-hospital (CASPRI) score.

^b^ Patients with cerebral performance score 1 or 2

^c^ VF, ventricular fibrillation

^d^ VT, ventricular tachycardia

^e^ CPR, cardiopulmonary resuscitation

^f^ CNS, central nervous system

As shown in Table 4, the CASPRI score was significantly associated with the primary outcome (OR: 0.83, 95% CI: 0.80–0.87); the AUROCC was 0.79 (95% CI: 0.74–0.84) (S2 Fig). The AUROCC did not change substantially when the CASPRI score was applied to two temporal subgroups, either ≦2010 or >2010.

**Table 4:** Performance of the CASPRI score as a predictive variable for favourable neurological outcome at hospital discharge.

Patient groups	Odds ratio (95% confidence interval)	Area under the ROC^b^ curve (95% confidence interval)
All patients	0.83 (0.80–0.87)	0.79 (0.74–0.84)
Patients receiving CPR^a^ during or before 2010	0.84 (0.79–0.90)	0.76 (0.66–0.86)
Patients receiving CPR after 2010	0.83 (0.79–0.87)	0.80 (0.74–0.85)

^a^ CPR, cardiopulmonary resuscitation

^b^ ROC, receiver operating characteristics

## Discussion

### Main findings

In this retrospective study conducted in a single centre at Taiwan, we identified the common prognosticators of IHCA shared by both East Asian and Western societies. As a composite prognosticator, the CASPRI score, derived from the GWTG-R database in the United States, was demonstrated to be significantly associated with IHCA outcome in an East Asian population. The AUROCC revealed that the CASPRI score had excellent accuracy for predicting the neurological outcome of patients resuscitated from IHCA in this population. The predictive performance was similarly accurate when the CASPRI score was applied to IHCA occurring after the 2010 resuscitation guidelines were implemented [11].

### Comparison with the original study

The mean age and the proportion of male were similar between our population and the GWTG-R cohort of the study reported by Chan et al. [6] The most striking difference between the studies may be the proportion with favourable neurological status prior to IHCA. In the study reported by Chan et al. [6], the proportion of pre-arrest favourable neurological status was 72.6%; in contrast, in our study, this proportion was only 43.6%. In the Taiwanese culture, the notion that “a bad life is better than a good death” is prevalent, which may explain why over half the patients in our study had unfavourable neurological status before CPR. Moreover, because withdrawal of life-sustaining therapy was illegal during the study period, the proportion of patients with neurologically intact survival in our study (11.8%) was less than half that of the GWTG-R cohort (24.6%). To relieve the significant burden on the family and society [3], a valid prediction model for outcomes of IHCA may be of great help in Taiwan.

### Comparison of prediction models regarding IHCA outcomes

To provide the treatment that best fits the patient’s preferences, physicians may attempt to discuss a plan for the details of CPR preemptively, including DNR orders, with the patients and/or their surrogates. However, Jones et al. [12] found that <50% of medical students, residents, and attending physicians were able to accurately assess the survival probability after IHCA. Therefore, many physicians may not be able to identify the target patient groups with whom they should discuss end-of-life choices. Less than 50% of inpatients who preferred not to receive CPR were reported to have written DNR orders [13]. Ebell et al. [14] used the GWTG-R database to develop the Good Outcome Following Attempted Resuscitation (GO-FAR) scoring system to identify patients who were unlikely to benefit from CPR after IHCA. The GO-FAR score, using pre-arrest variables, may serve as the basis for the patient, family and clinicians to discuss the CPR choices upon admission before IHCA actually occurs.

However, the unique East Asian culture may hinder application of the GO-FAR score. In a survey of Taiwanese physicians, Cheng et al. [5] demonstrated that 70% often or very often observed reluctance on the part of family members to discuss end-of-life issues. This phenomenon was also observed in Japan and Korea; the corresponding frequencies were 59% in Korea and 50% in Japan. In these East Asian areas, about 50% of physicians reported that the common belief “bad things happen after you say them out loud” might lead to this reluctance [5]. For patients with definite terminal diseases, such as incurable cancer, physicians could more easily present the option of DNR orders to patients and their family. However, for patients with other acute critical comorbidities, such as pneumonia with respiratory insufficiency or septic shock, it would be difficult for physicians to discuss CPR plans pre-emptively before IHCA occurred even if the estimated probabilities of favourable outcomes following IHCA were low. Therefore, although the GO-FAR score [14] is a useful tool to identify patients with an extremely low survival probability following IHCA, its use may be difficult in this cultural setting. Considering the local culture, we thought that the CASPRI score may be more appropriate in application since this score could provide concrete probabilities for favourable neurological survival after patients achieved ROSC following IHCA.

### Validation of CASPRI score in a Taiwanese cohort

To construct the prediction model, we included variables with the same definitions reported by Chan et al. [6] in our regression analysis. Six of the twelve variables in our final model, CPR duration, neurological status before IHCA, malignant disease, initial arrest rhythms, renal insufficiency and age, were also components of the CASPRI score. In the study reported by Chan et al. [6], CPR duration and age were analysed as ordinal variables. Because we enrolled far fewer patients than did the study reported by Chan et al. [6], we analysed these two factors as continuous variables. The results were similar, i.e., patients with longer the CPR duration or older age were less likely to survive with favourable neurological status. In our model, depression in central nervous system function and myocardial infarction during this admission were shown to be significantly associated with the outcome. Although these two variables were not components of the final CASPRI score, during the development of the CASPRI score by Chan et al. [5], these two variables were included in the initial model and then dropped from the final model in the pursuit of model parsimony. Thus, eight of 12 variables used in our model were also identified by Chan et al. [5] while developing the CASPRI score even though our study cohort was enrolled from a medical centre in Taiwan, which had many differences from the GWTG-R cohort in the United States.

By using GWTG-R database, Chan et al. [7] and Razi et al. [8] also demonstrated that the IHCA outcomes differ according to ethnicity. Black patients were significantly less likely to survive to discharge than white patients, regardless of whether the initial arrest rhythm was ventricular fibrillation/pulseless ventricular tachycardia [7] or pulseless electrical activity/ asystole [8]. In these two studies [7, 8], the proportion of Asian patients was low and Asian patients were excluded from analysis; therefore, the influence of Asian ethnicity on IHCA outcomes was unknown. Chan et al. [6] did not report the proportion of Asian patients in the GWTG-R cohort used for developing the CASPRI score, but the proportion could be low according to two previous studies [7, 8] that analysed the GWTG-R database to evaluate the effect of ethnicity on IHCA outcomes. Accordingly, the generalizability of the CASPRI score to an East Asian population should be further examined. Our results demonstrated that the accuracy of the CASPRI score applied in our cohort was excellent; the AUROCC was 0.79, similar to the AUROCC (0.80) achieved when the CASPRI score was internally validated in the original study reported by Chan et al. [6]. Moreover, the CASPRI score was developed using the GWTG-R cohort enrolled between 2000 and 2009. In our regression model, IHCA during or before 2010 was shown to be inversely associated with favourable neurological outcome. Therefore, the generalizability of the CASPRI score to IHCA patients after the implementation of 2010 resuscitation guidelines [11] was also examined. The results indicated that when the CASPRI score was applied to patients with IHCA that occurred after year 2010, the accuracy was similarly excellent; the AUROCC was 0.80.

In summary, our validation study demonstrated that the CASPRI score could be applied to an East Asian population with excellent accuracy, which was not modified by the time period. To better emphasize personalised medicine, providing quantified probabilities for neurologically intact survival for patients achieved ROSC following IHCA would greatly help to manage the expectations of the patients and their family. This may be especially important in an East Asian society because the family may define medical futility very differently from the healthcare staff. With concrete estimates, it may be easier for patients and their family to realise the prognosis and make critical decisions.

### Study limitations

First, a major limitation of this study was the retrospective design. The CPC score was not routinely measured during hospitalization. However, at NTUH, the nursing staff assesses the level of consciousness every shift and records basic activities of daily living on admission, every shift, before change of care unit and hospital discharge. Basic activities of daily living consist of self-care tasks, including functional mobility, bathing and showering, dressing, self-feeding, personal hygiene and grooming, and toilet hygiene. Also, nursing records were detailed and comprehensive, sometimes even including the dialogue between the patients and primary care nurses. Through these nursing records, the CPC score was retrospectively determined with some accuracy. Even though misclassification bias probably occurred, the bias should be nondifferential, which should not affect the validity of our study results. Second, a successful validation of CASPRI score in our cohort might not promise its prognostic accuracy in other populations with different characteristics. Further large-scale validation studies should be performed to prove its universal applicability.

## Conclusions

In this retrospective study conducted in a single centre at Taiwan, we identified the common prognosticators of IHCA shared by both East Asian and Western societies. As a composite prognosticator, the CASPRI score was shown to predict neurological outcome with excellent accuracy among successfully resuscitated IHCA patients in an East Asian population. The predictive accuracy performed similarly well when the CASPRI score was used to predict IHCA outcomes during two different time periods. This tool allows accurate IHCA prognostication for physicians, patients, and families in an East Asian population that should improve discussions of treatment plans.

## Supporting information

S1 Table Cardiac Arrest Survival Postresuscitation In-hospital (CASPRI) score. (DOCX)

S1 Fig Flowchart of the patient inclusion process. (TIFF)

S2 Fig Receiver operating characteristics curve of the CASPRI score to predict the favourable neurological outcome at hospital discharge. (TIF)

S1 Dataset Raw data used in statistical analysis. (XLSX)
