# Supplementary material for: Validation of the Cardiac Arrest Survival Postresuscitation In-hospital (CASPRI) score in an East Asian population
Source: PLoS One. 2018 Aug 23;13(8):e0202938. doi: 10.1371/journal.pone.0202938 (PMC6107241; doi:10.1371/journal.pone.0202938)
Supplement: S1 Table — (DOCX) [file pone.0202938.s001.docx]

S1 Table. Cardiac Arrest Survival Postresuscitation In-hospital (CASPRI) Score

| Predictor | Points |
| --- | --- |
| 1. Age group, y |  |
| <50 | 0 |
| 50-59 | 0 |
| 60-69 | 1 |
| 70-79 | 2 |
| ≧80 | 4 |
| 1. Initial arrest rhythm |  |
| VF^a^/VT^b^ time to defibrillation |  |
| ≦2 minutes | 0 |
| 3 minutes | 0 |
| 4-5 minutes | 2 |
| >5 minutes | 3 |
| Pulseless electrical activity | 6 |
| Asystole | 7 |
| 1. Prearrest CPC^c^ score |  |
| 1 | 0 |
| 2 | 2 |
| 3 | 9 |
| ≧4 | 9 |
| 1. Hospital location |  |
| Telemetry unit | 0 |
| Intensive care | 1 |
| Nonmonitored unit | 3 |
| 1. Duration of resuscitation, minutes |  |
| <2 | 0 |
| 2-4 | 0 |
| 5-9 | 3 |
| 10-14 | 5 |
| 15-19 | 6 |
| 20-24 | 6 |
| 25-29 | 6 |
| ≧30 | 8 |
| Factors present prior to arrest |  |
| 1. Mechanical ventilation | 3 |
| 1. Renal insufficiency | 2 |
| 1. Hepatic insufficiency | 4 |
| 1. Sepsis | 3 |
| 1. Malignant disease | 4 |
| 1. Hypotension | 3 |

^a^ VF, ventricular fibrillation

^b^ VT, ventricular tachycardia

^c^ CPC, cerebral performance score
