# Supplementary figures and images for: Validation of the Cardiac Arrest Survival Postresuscitation In-hospital (CASPRI) score in an East Asian population
Source: PLoS One. 2018 Aug 23;13(8):e0202938. doi: 10.1371/journal.pone.0202938 (PMC6107241; doi:10.1371/journal.pone.0202938)

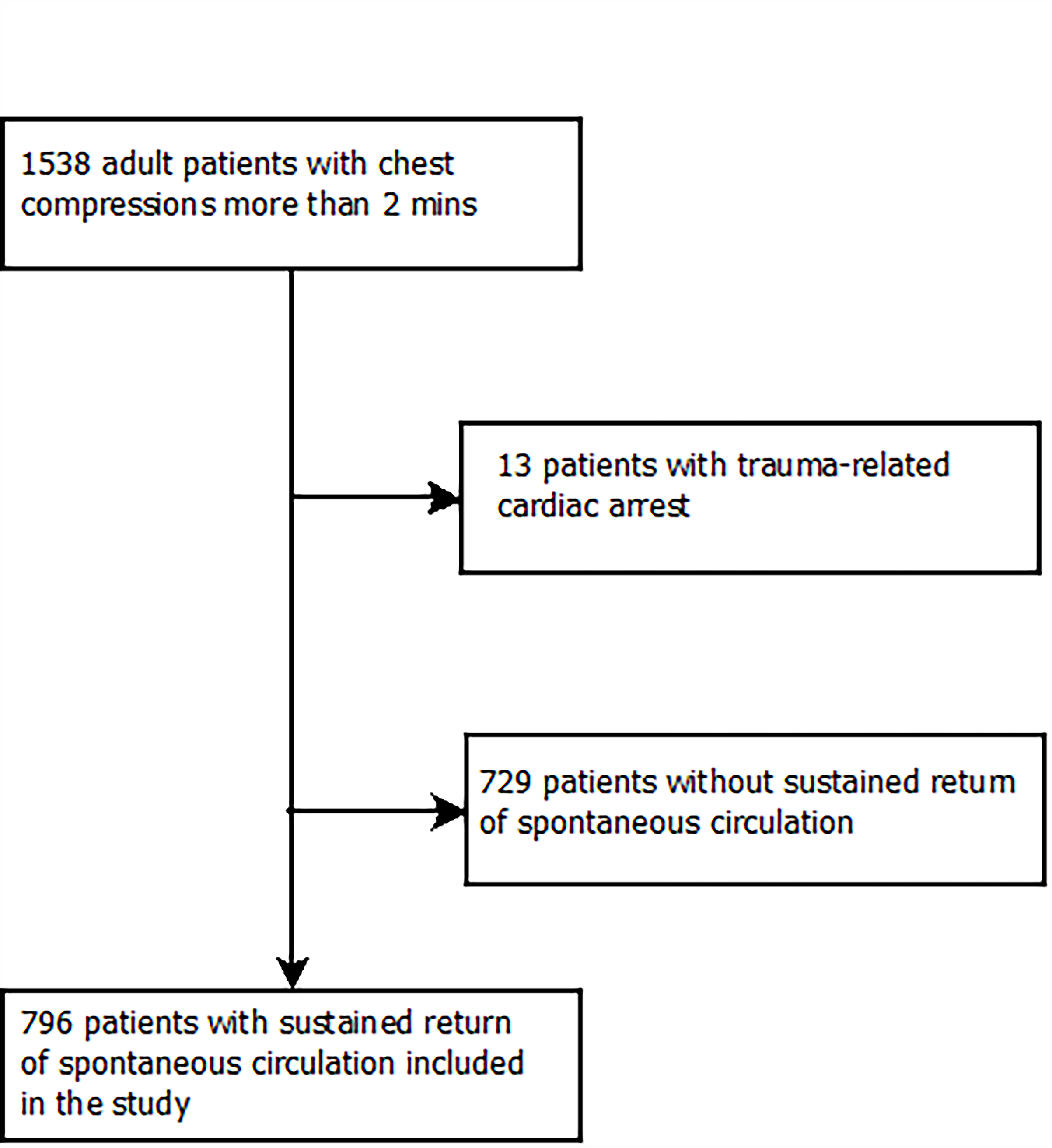

Supplement: S1 Fig — (TIFF) [file pone.0202938.s002.tiff]

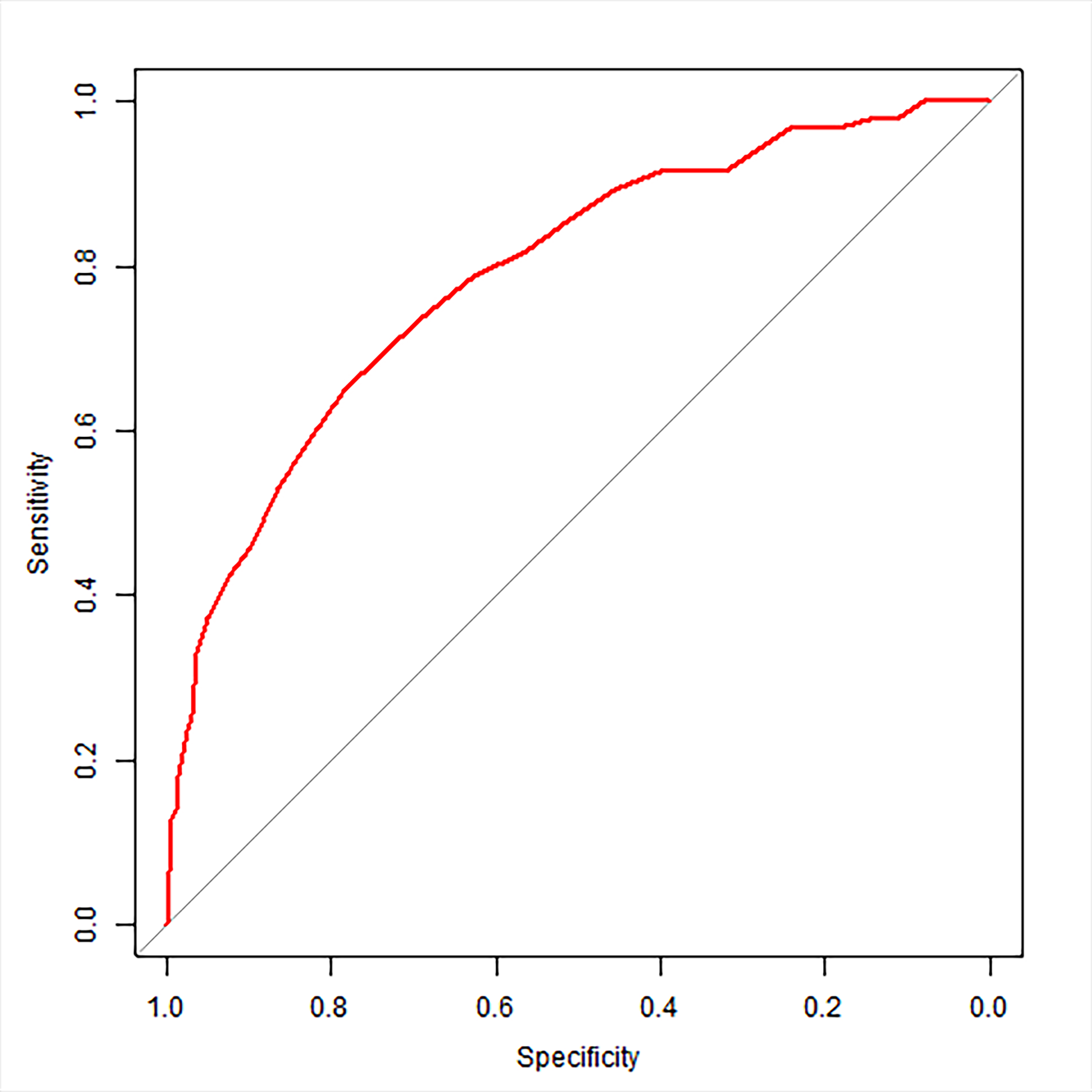

Supplement: S2 Fig — (TIF) [file pone.0202938.s003.tif]
